# Role of lncRNAs in acute pancreatitis: pathogenesis, diagnosis, and therapy

**DOI:** 10.3389/fgene.2023.1257552

**Published:** 2023-09-28

**Authors:** Jie Deng, Ziying Song, Xiaolan Li, Huiqing Shi, Shangqing Huang, Lijun Tang

**Affiliations:** ^1^ Department of Clinical Pharmacy, The General Hospital of Western Theater Command, Chengdu, China; ^2^ Department of Emergency Medicine, The General Hospital of Western Theater Command, Chengdu, China; ^3^ Department of Pain Medicine, The General Hospital of Western Theater Command, Chengdu, China; ^4^ Department of General Surgery, Pancreatic Injury and Repair Key Laboratory of Sichuan Province, The General Hospital of Western Theater Command, Chengdu, China; ^5^ School of Materials Science and Engineering, Southwest Jiaotong University, Chengdu, China

**Keywords:** acute pancreatitis, lncRNAs, pathogenesis, diagnosis, therapy

## Abstract

Acute pancreatitis (AP) is one of the most common acute abdominal diseases characterized by an injury and inflammatory disorder of the pancreas with complicated pathological mechanisms. Long non-coding RNAs (lncRNAs) have been shown to play an important role in various physiological and pathological processes in humans, and they have emerged as potential biomarkers of diagnosis and therapeutic targets in various diseases. Recently, accumulating evidence has shown significant alterations in the expression of lncRNAs, which are involved in the pathogenesis of AP, such as premature trypsinogen activation, impaired autophagy, inflammatory response, and acinar cell death. Moreover, lncRNAs can be the direct target of AP treatment and show potential as biomarkers for the diagnosis. Thus, in this review, we focus on the role of lncRNAs in the pathogenesis, diagnosis, and therapy of AP and emphasize the future directions to study lncRNAs in AP, providing new insight into understanding the cellular and molecular mechanisms of AP and seeking novel biomarkers for the diagnosis and therapeutic targets to improve clinical management in the future.

## 1 Introduction

Acute pancreatitis (AP) is a common acute abdominal disease characterized by injury and inflammatory disorder of the pancreas (Lee and Papachristou, 2019). In patients with AP, approximately 80% have mild and moderate symptoms, but 20% of them develop severe AP (SAP). Due to massive necrosis of the pancreatic tissue, systemic inflammatory response syndrome (SIRS), persistent organ failure, and subsequent infection, the mortality rate of patients can be as high as 30%, leading to serious medical and social burdens worldwide (Garg and Singh, 2019; Lee and Papachristou, 2019; Trikudanathan et al., 2019). The main causes of AP include gallstones, alcohol, hyperlipidemia, some drugs, and endoscopic retrograde cholangiopancreatography (Harvey et al., 1989; Mederos et al., 2021), which can result in an increase in ductal pressure, interstitial edema, accumulation of enzyme-rich fluid within the pancreatic tissues, and disruption of multiple biochemical pathways within acinar cells (Harvey et al., 1989; Lee and Papachristou, 2019; Mederos et al., 2021). In the development of AP, a series of pathogenesis can be activated simultaneously or subsequently, such as premature trypsinogen activation, mitochondrial dysfunction, impaired autophagy, inflammatory response, and endoplasmic reticulum stress, thereby leading to SIRS and aggravating pancreatic injury (Jakkampudi et al., 2016; Biczo et al., 2018; Lee and Papachristou, 2019; Mederos et al., 2021). These pathological events, SIRS, and organ failure are interrelationships, resulting in complicated pathological mechanisms in AP (Garg and Singh, 2019; Lee and Papachristou, 2019; Ferrero-Andrés et al., 2020; Sendler et al., 2020). However, the main treatment is conservative treatment or operation in a clinical setting, and early management lacks curative options. Thus, it is imperative to understand the pathogenesis of AP, in order to develop new diagnostic markers and therapeutic targets for novel intervention strategies in clinical settings.

Long non-coding RNAs (lncRNAs) are a type of RNA longer than 200 nucleotides first reported in 1990 (Herman et al., 2022). For a long time after that, lncRNAs have been considered “noise sequences” (Necsulea et al., 2014). With the development of high-throughput sequencing and bioinformatics, a number of studies have revealed the features and functions of lncRNAs (Quinn and Chang, 2016). Despite their similarities to mRNAs, which are transcribed by RNA polymerase II and are capped, polyadenylated, and spliced, lncRNAs have their unique features and functions (Chen, 2016; Quinn and Chang, 2016). The function of lncRNAs depends on their subcellular localization, and many lncRNAs exhibit distinct nuclear localization patterns (Chen, 2016). At different subcellular localizations, lncRNAs can regulate physiological and pathological cellular activities by genomic expression modulation, epigenetic modification, and post-transcriptional regulation in cis or in trans by interacting with chromatins, proteins, and RNAs in the nucleus or cytoplasm (Chen, 2016). LncRNAs are aberrantly expressed and found to be involved in the occurrence and development of many diseases, such as cancers (Tan et al., 2021), cardiovascular diseases (Huang, 2018), neurological diseases (Ma et al., 2022), and intestinal diseases (Chen et al., 2018). In recent years, lncRNAs have been found to play critical roles in the molecular mechanism, diagnosis, and therapy of AP.

Therefore, in this review, we focus on the role of lncRNAs in the pathogenesis, diagnosis, and therapy of AP and emphasize the future directions to study lncRNAs in AP, providing new insight into understanding the cellular and molecular mechanisms of AP and seeking novel biomarkers for the diagnosis and therapeutic targets to improve clinical management in the future.

## 2 Aberrantly expressed lncRNAs in AP

Recently, many studies have shown that the expression of lncRNAs is more specific than that of mRNAs by next-generation sequencing and presents a cell type-, tissue-, developmental stage-, or disease state-specific manner (Mercer et al., 2008; Batista and Chang, 2013; Flynn and Chang, 2014), suggesting that lncRNAs may be involved in different pathophysiological processes. In AP, several studies have reported the aberrantly expressed lncRNAs in patients and the rat/mice AP model (Xia et al., 2020; Wang et al., 2021; Liu H. et al., 2022). For example, in the whole peripheral blood from patients with AP and recurrent AP, 1,065 and 409 differentially expressed lncRNAs compared with normal controls in AP and recurrent AP were identified by RNA-seq, respectively (Liu H. et al., 2022). There were 315 lncRNAs expressed both in AP and recurrent AP, and their functions may be regulating the MAPK signaling pathway and metabolic pathways, suggesting that these lncRNAs may be involved in AP development and recurrence. In the rat model of AP, Xia et al. identified 1,156 distinctively dysregulated lncRNAs, and these dysregulated lncRNAs are associated with the immune system process by bioinformatics analysis (Xia et al., 2020). In a mice model of SAP, 4,459 lncRNAs were identified and differentially expressed lncRNAs are mainly involved in the apoptosis pathway and calcium signal transduction pathway. These studies indicate the important role of lncRNAs in AP (Wang et al., 2021). However, data based on high-throughput sequencing lack conjoint analysis among different studies and the roles of a large number of lncRNAs in AP remain unclear.

## 3 LncRNAs in AP pathogenesis

**FIGURE 1:**
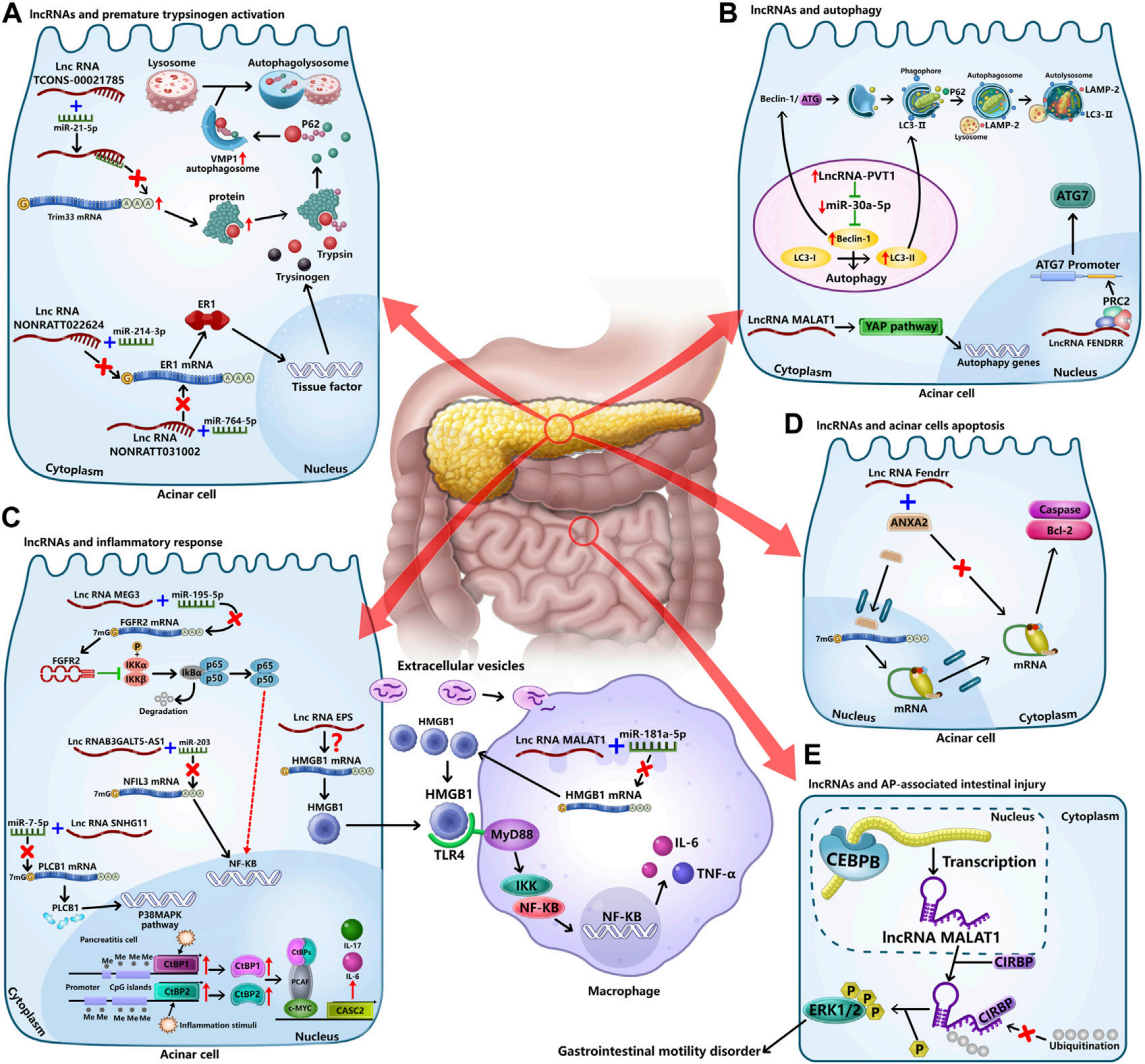
Roles of lncRNAs in AP pathogenesis. Based on the current reports, the studies about the roles of lncRNAs in AP pathogenesis mainly focused on four aspects, including premature trypsinogen activation, impaired autophagy, inflammatory response, and acinar cells death. **(A)** LncRNA TCONS_00021785 upregulates the expression of E3 ligase enzyme Trim33 by binding miR-21-5p to regulate VMP1-mediated zymophagy, reducing the activation of trypsinogen. LncRNA NONRATT022624 and lncRNA NONRATT031002 increase the expression of transcription factor ER1 by binding miR-214-3p and miR-764-5p, regulating the expression of tissue factor and enhancing trypsinogen activation. **(B)** LncRNA PVT1 and lncRNA FENDRR influence the initial stage of autophagy. Mechanistically, lncRNA PVT1 upregulates the expression of beclin1 by binding miR-30a-5p to induce the abnormal autophagy, and lncRNA FENDRR inhibits the expression of ATG7 via binding to RNA-binding protein PRC2 which can bind to promoter regions of ATG7. LncRNA MALAT1 inhibits of autophagy by upregulating YAP, but the specific mechanism remains unclear. **(C)** In inflammatory response of AP, lncRNAs mainly regulate the NF-κB pathway by direct or indirect ways, inducing the expression and release of inflammatory factors and M1 polarization of macrophages. **(D)** LncRNA Fendrr binds to ANXA2 protein directly to promote acinar cell apoptosis. ANXA2 protein, mostly in the nucleus, can bind to the 3′-UTRs of the specific mRNAs to inhibit its translation. **(E)** Transcription factor CEBPB can bind to the promoter region of lncRNA MALAT1 and promote its expression. LncRNA MALAT1 interacts with CIRBP and inhibits its ubiquitination, resulting in the activation of the ERK pathway and the gastrointestinal motility disorder. However, the roles of lncRNAs in AP-associated organ injury, including lung injury, liver injury, myocardial injury, and kidney injury, are unclear.

Although the exploration of lncRNAs in AP is less than 5 years and the function of a large number of dysregulated lncRNAs in AP remains unclear, some studies have shown that lncRNAs play important roles in AP pathogenesis (Table 1; Figure 1). In this section, we focused on the roles of lncRNAs in AP pathogenesis based on current reports.

**TABLE 1:** Role of lncRNAs in AP pathogenesis.

LncRNAs	Expression	Pathological event	Role	References
LncRNA TCONS_00021785	Up	Premature trypsinogen activation	Protecting acinar cells by sponging miR-21-5p to regulate pancreatin ubiquitination and trypsinogen activation	Wang et al. (2022)
LncRNA NONRATT022624	Up	Premature trypsinogen activation	Enhancing trypsinogen activation by sponging miRNA-214-3p to increase the expression of early growth response protein 1	Gao et al. (2020)
LncRNA NONRATT031002	Up	Premature trypsinogen activation	Enhancing trypsinogen activation by sponging miR-764-5p to increase the expression of early growth response protein 1	Gao et al. (2020)
LncRNA FENDRR	Up	Autophagy	Inhibited the expression of ATG7 via binding to PRC2 (a RNA-binding protein), thereby inducing the aberrant autophagy, promoting the injury of acinar cells	Zhao et al. (2021)
LncRNA-PVT1	Up	Autophagy	Inducing the abnormal autophagy by binding miR-30a-5p to upregulate the expression of Beclin-1 and LC3-II	Hernández et al. (2020)
LncRNA MALAT1	Up	Autophagy	Aggravating pancreatic cell inflammation via the inhibition of autophagy by upregulating YAP	Liu et al. (2022)
LncRNA NEAT1	Up	Inflammatory response	Increasing the release of IL-6 and TNF-α by sponging miR-365a-3p to induce inflammatory response	Shao et al. (2022)
LncRNA CASC2	Up	Inflammatory response	Activating the expression of IL-6 and IL-17 and aggravating the inflammation response of AP.	Zeng et al. (2020)
LncRNA MEG3	Down	Inflammatory response	Inhibiting cell apoptosis and inflammatory responses via regulating the miR-195-5p/FGFR2 axis and NF-κB pathway	Chen et al. (2021)
LncRNA B3GALT5-AS1	Down	Inflammatory response	Alleviating acinar cell injury by inhibiting the activation of the NF-kB pathway	Wang et al. (2018)
LncRNA SNHG11	Down	Inflammatory response	Regulating phospholipase C beta 1(PLCB1) by sponging miR-7-5p to reduce the p38MAPK expression	Song et al. (2022)
LncRNA MALAT1	Up	Inflammatory response	1) Upregulating the expression of HMGB1 to activate the TLR4/NF-κB pathway through binding to miR-181a-5p, thereby facilitating the M1 polarization of macrophages, ultimately promoting the occurrence and progression of AP. 2) Increasing the levels of IL-6 and TNF-α by regulating the Hippo-YAP signaling pathway	Hou et al. (2020); Huang et al. (2018); Liu et al. (2021)
LncRNA EPS	Down	Inflammatory response	Suppressing HMGB1-triggered inflammation in pancreatic macrophages	Chen et al. (2021)
LncRNA Fendrr	Up	Acinar cell death	Promoting AR42J cell apoptosis by directly binding ANXA2 protein	Zhao et al. (2019)

### 3.1 LncRNAs and premature trypsinogen activation

As we all know, premature trypsinogen activation is an important pathological event at the early stage of initiating AP (Lerch and Gorelick, 2000; Dawra et al., 2011). Under the action of injury factors, zymogen particles are transported to lysosomes, which result in their fusion in acinar cells. Once the fusion of zymogen particles and lysosomes takes place, the lysosomal enzyme cathepsin B will activate trypsinogen to trypsin (Halangk et al., 2000; Dawra et al., 2011). Once activated and released, trypsin will cause autodigestion of the acinar cells and adjacent tissues, leading to necrosis and inflammatory response (Dawra et al., 2011; Talukdar et al., 2016). Thus, elucidating the mechanisms of occurrence and regulation of premature trypsinogen activation is key to seeking therapeutic targets for inhibiting the development of AP.

LncRNAs can regulate the activation of trypsinogen. For example, zymophagy, a type of selective autophagy, can reduce the activation of pancreatin at the early stage of AP, thus maintaining cell homeostasis and the function of pancreatic exocrine secretion as the defense mechanism (Grasso et al., 2011; Mareninova et al., 2020). Recently, Wang et al. (2022) found that lncRNA TCONS_00021785 was important to inhibit the activation of trypsinogen by regulating zymophagy. At this progress, the E3 ligase enzyme Trim33 was found to play a critical role. In AR42J cells treated with taurolithocholic acid 3-sulfate (TLCS), the overexpression of Trim33 could reduce cell necrosis and trypsinogen activation via mediating trypsin ubiquitination to initiate the zymophagy by regulating the expression of VMP1. Further study found that miR-21-5p was critical to control the expression of Trim33. Based on lncRNA microarray and bioinformatic analysis, they found that lncRNA TCONS_00021785 could bind to miR-21-5p. After silencing lncRNA TCONS_00021785, the expression of miR-21-5p was significantly increased, the expression of Trim33 was significantly reduced, and the activation of zymogen was remarkably increased. These findings showed the important role of lncRNA TCONS_00021785 in protecting acinar cells by reducing zymogen activation. In another study, Gao B. et al. (2020) found that lncRNA NONRATT022624 and NONRATT031002 could regulate the expression of early growth response protein 1 (Egr1, a transcription factor) by binding miR-214-3p and miR-764-5p, resulting in increased trypsinogen activation. These studies suggested that lncRNAs may play a key role in trypsinogen activation. Nevertheless, further studies are needed to explore the specific mechanisms.

### 3.2 LncRNAs and autophagy

Autophagy is a cytoprotective mechanism that recycles and processes damaged, defective, and aged cytoplasmic contents. For example, selective macro-autophagy can process specific damaged organelles and misfolded proteins (Antonucci et al., 2015; Gukovskaya et al., 2017). Autophagy begins with the enucleation of cytosolic contents in an open double membrane formed with the ER, Golgi apparatus, and plasma membrane (Antonucci et al., 2015; Gukovskaya et al., 2017). Under the action of autophagy-related proteins (ATGs), the double membrane edges form an autophagosome. In AP, impaired autophagy can cause trypsinogen activation, inflammation, ER stress, and mitochondrial dysfunction (Antonucci et al., 2015; Gukovskaya et al., 2017; Biczo et al., 2018; Zelic et al., 2018), ultimately leading to the death of acinar cells. Therefore, it is very important for acinar cells to produce proteins efficiently.

Recent studies have shown that lncRNAs can regulate autophagy from different aspects of AP. For example, Zhao et al. (2021) observed that lncRNA FENDRR, a lncRNA that regulates many gastrointestinal diseases, was upregulated and was associated with apoptosis and aberrant autophagy in cell models and mouse models of AP. Mechanistically, lncRNA FENDRR inhibited the expression of ATG7 via binding to PRC2 (an RNA-binding protein), thereby inducing aberrant autophagy, and promoting injury of acinar cells. In SAP, lncRNA PVT1 was significantly upregulated and could bind miR-30a-5p, upregulating the expression of Beclin-1 and LC3-II and leading to the abnormal autophagy, suggesting that lncRNA PVT1 aggravated SAP via promoting autophagy by the miR-30a-5p/Beclin-1 axis (Hu et al., 2020). In addition, in non-alcoholic fatty liver disease, damaged hepatocytes produced lots of exosomes (Hernández et al., 2020), promoting inflammatory responses in pancreatic acinar cells. Mechanistically, the exosome-derived lncRNA MALAT1 was upregulated significantly and inhibited the hippo-YAP pathway and autophagy in AP. The YAP1 inhibitor CA3 could increase the LC3II/LC3I expression and reduce IL-6 and TNF-α levels induced by lncRNA MALAT1, suggesting that lncRNA MALAT1 aggravated AP by suppressing autophagy via the YAP pathway (Liu D. et al., 2022). However, the specific mechanism of the YAP pathway to regulate autophagy in AP is unclear.

Interestingly, the regulatory mechanisms of lncRNAs are very important to understanding their gene regulation function during the development of various diseases. Recent studies reported that N6-methyladenosine (m6A) modification can regulate the function of lncRNAs by various mechanisms, including their stability, subcellular localization, interactions with proteins, miRNAs, and mRNA, and regulation of gene transcription (He et al., 2020). In AP, a recent study reported three m6A-related lncRNAs (lncRNA Pvt1, lncRNA Meg3, and lncRNA AW112010) may regulate eight autophagy genes to affect the development of AP through constructing lncRNA–miRNA–mRNA networks (Li et al., 2022). Although this study did not provide experimental verification, it suggested that it is essential to explore the role of m6A-modified lncRNAs in AP, based on the important role of m6A in lncRNA function.

### 3.3 LncRNAs and inflammatory response

An inflammatory response is the pivotal pathogenesis for AP to develop SAP (Garg and Singh, 2019; Lee and Papachristou, 2019). At the early stage of AP, injured acinar cells release different inflammatory mediators, such as cytokines and chemokines, which recruit inflammatory cells into injured sites, leading to the local sterile inflammatory response (Jakkampudi et al., 2016; Watanabe et al., 2017; Ferrero-Andrés et al., 2020). If the local inflammation cannot be controlled, SIRS can occur and result in multiple organ failure (Garg and Singh, 2019). During this pathological process, multiple signaling pathways are activated in acinar cells and inflammatory cells, such as TLR4/NF-κB pathways (Jakkampudi et al., 2016; Li et al., 2016), JAK/STAT pathways (Komar et al., 2017), TGF-β/SMAD pathways (Riesle et al., 1997), and MAPK signaling pathway (Zhou et al., 2019). Thus, it is essential to understand the specific mechanisms of inflammation response to inhibit AP development. In recent years, given the important roles of lncRNAs in regulating immune response, some studies have explored the role of lncRNAs in this pathogenesis of AP.

Injured acinar cells release pro-inflammatory cytokines, such as IL-6, IL-17, and TNF-α, that can initiate the inflammatory response. Some lncRNAs increase the inflammatory injury of acinar cells. For example, Shao et al. (2022) found that lncRNA NEAT1 was upregulated in the AR42J cells treated with caerulein and can increase the release of IL-6 and TNF-α by sponging miR-365a-3p. The inhibition of the expression of lncRNA NEAT1 decreased the apoptosis and inflammation of the AR42J cells. In another study, Zeng et al. (2020) performed a microarray analysis and found 21 differentially expressed lncRNAs from pancreatic tissues of AP patients and identified that lncRNA CASC2 induced the increased expression of IL-6 and IL-17. In a further study, they found that transcription factor c-MYC recruited transcription coactivators PCAF and transcription corepressors CtBP1/2 to the promoter of lncRNA CASC2 and activated its expression. The CtBP-associated transcriptional complex was activated by both DNA methylation and inflammatory stimuli. This study suggested that DNA methylation and inflammatory stimuli can co-regulate the CtBP-PCAF-c-MYC transcriptional complex to activate the expression of lncRNA CASC2, activating the expression of IL-6 and IL-17, aggravating inflammatory response in AP.

As we all know, NF-κB is a ubiquitous transcription factor that is very important for the production of inflammatory cytokines in immune response (Jakkampudi et al., 2016). It has been reported that NF-κB is activated aberrantly in the early stage of AP and is pivotal for inflammatory cytokine expression (Vaquero et al., 2001; Jakkampudi et al., 2016). Recent studies found that lncRNAs participated in the inflammatory response of AP by regulating the NF-κB pathway. LncRNA MEG3 participated in the pathogenesis of many diseases. Chen and Song (2021) found that the expression of lncRNA MEG3 was reduced and the overexpression of lncRNA MEG3 can inhibit cell apoptosis and inflammatory responses of AP. Mechanically, miR-195-5p is the downstream target of lncRNA MEG3, and miR-195-5p can bind to fibroblast growth factor receptor 2 (FGFR2). In caerulein-induced human pancreatic duct epithelial cells, the intervention of lncRNA MEG3, miR-195-5p, or FGFR2 and the expression of NF-κB pathway proteins were altered significantly, suggesting that lncRNA MEG3 participated in AP via regulating the miR-195-5p/FGFR2 axis and NF-κB pathway. However, the specific relationship between lncRNA MEG3 and the NF-κB pathway is unclear. LncRNA B3GALT5-AS1 was observed first in colon cancer tissues (Wang et al., 2018). In AP, the expression of lncRNA B3GALT5-AS1 is reduced and the overexpression of lncRNA B3GALT5-AS1 can alleviate the injury of acinar cells (Wang et al., 2019). Mechanically, lncRNA B3GALT5-AS1 regulated the expression of nuclear factor interleukin-3 (NFIL3) by sponging miR-203. In addition, the overexpression of lncRNA B3GALT5-AS1 can reduce the levels of p50 and p65, indicating that lncRNA B3GALT5-AS1 alleviates acinar cell injury by inhibiting the activation of the NF-kB pathway. In other important signaling pathways for regulating inflammatory response, the expression of lncRNA SNHG11 in AP was reduced and overexpression could inhibit apoptosis and inflammatory responses (Song et al., 2022). Mechanically, lncRNA SNHG11 regulated Phospholipase C Beta 1(PLCB1) by sponging miR-7-5p. The overexpression of lncRNA SNHG11 and PLCB1 could reduce the p38MAPK expression, indicating that the overexpressed lncRNA SNHG11/miR-7-5p/PLCB1 axis inhibited AP progression via regulating the p38MAPK signaling pathway.

In addition to acinar cells, the macrophage is the major inflammatory cell that participates in the inflammatory response of AP (Gea-Sorlí and Closa, 2010). Accumulating evidence has proved that lncRNA MALAT1 plays an important in the M1/M2 subtypes of macrophages (Huang et al., 2018; Hou et al., 2020). In AP, Liu et al. (2021) found that EV-encapsulated lncRNA MALAT1 could upregulate the expression of HMGB1 and activate the TLR4/NF-κB pathway through binding to miR-181a-5p, thereby facilitating the M1 polarization of macrophages, ultimately promoting the occurrence and progression of AP. As a well-known lncRNA, lncRNA MALAT1 can also be involved in the inflammation of AP by other mechanisms. The Yes-associated protein 1 (YAP1) transcriptional coactivator is a prime mediator of the Hippo signaling pathway, and the Hippo-YAP signaling pathway is involved in regulating cell proliferation, migration, and tissue regeneration (Lian et al., 2010). Gu et al. (2019) reported that lncRNA MALAT1 can bind to miR-194, thereby regulating the expression of YAP1. The overexpression of MALAT1 or YAP1 can increase the levels of IL-6 and TNF-α, indicating that lncRNA MALAT1 can influence the progression of AP by regulating the Hippo-YAP signaling pathway. In addition to lncRNA MALAT1, lncRNA EPS was also found to be involved in AP by regulating pancreatic macrophages. In the model of caerulein-induced AP and sodium taurocholate-induced SAP, the expression pattern of lncRNA EPS is negatively correlated with inflammatory factors, IL-6, IL-1β, CXCL1, and CXCL2, and lncRNA EPS protects against injury of other organs, such as the liver, gut, and lungs (Chen et al., 2021). Inhibiting the activity of NF-kB in macrophages can abolish the suppressive effect of lncRNA EPS on TLR4 ligand-induced inflammatory genes, suggesting that lncRNA-EPS can alleviate SAP by suppressing inflammation in pancreatic macrophages.

### 3.4 LncRNA and acinar cell death

In the progress of AP occurrence and development, different acinar cell deaths can occur, such as apoptosis, necrosis, and pyroptosis (Bhatia, 2004). Acinar cell death will lead to the release of pancreatic enzymes and inflammatory mediators, ultimately exacerbating AP (Trikudanathan et al., 2019; Gao et al., 2021). Thus, exploring the specific mechanism of acinar cell death can help seek potential targets. Zhao et al. (2019) reported that LncRNA Fendrr was also upregulated in AP and promoted AR42J cell apoptosis by directly binding the annexin A2 protein. The annexin A2 protein is a calcium-dependent phospholipid-binding protein that participates in multiple biological processes, such as signal transduction, DNA synthesis, cell proliferation, and apoptosis (Vedeler et al., 2012; Chu et al., 2013), and it was suggested that lncRNAs play an important role in acinar cells death. However, currently, studies reporting the role of lncRNAs in acinar cell death are lacking. In the future, more studies should focus on the lncRNAs in acinar cell death.

Based on the aforementioned analysis, we can confirm that lncRNAs take part in the pathogenesis of AP from different aspects. However, their roles in other pathological events, such as calcium signaling, endoplasmic reticulum stress, ductal cell dysfunction, and intraductal events, are unclear. This is an important direction to explore the pathogenesis of AP in the future.

## 4 LncRNAs are involved in AP-associated organ injury

Organ injury is a common complication of AP, especially SAP, including intestinal injury, lung injury, liver injury, myocardial injury, and kidney injury. Of them, intestinal injury plays a critical role in AP progression. The injury of the intestinal mucosa barrier can induce intestinal bacterial translocation, which is the main cause of pancreatic necrotic tissue infection and ultimately leads to SIRS and multiple organ dysfunction syndrome (Cen et al., 2018). Chen et al. (2021) observed that lncRNA EPS can protect extra-pancreatic organs, such as the lungs, liver, and gut, from damage by suppressing the inflammation triggered by HMGB1. However, the specific mechanisms remain unclear. Lai et al. (2023) found that lncRNA MALAT1 was highly expressed in the plasma of SAP patients and pancreatic and intestinal tissues in the mice model of SAP, and knockdown of lncRNA MALAT1 could alleviate pancreatic and intestinal injury. Mechanistically, the transcription factor CEBPB was highly expressed in pancreatic and intestinal tissues and bound to the promoter region of lncRNA MALAT1, thereby activating the transcription of MALAT1. Activated lncRNA MALAT1 could interact with CIRBP [an RNA-binding protein involved in regulating inflammatory response (Lee et al., 2016)] and inhibit its degradation, resulting in the activation of the extracellular signal-regulated kinase (ERK) pathway, ultimately leading to gastrointestinal motility dysfunction in the mice of SAP. These two studies suggested that lncRNAs may play a critical role in AP-associated organ injury. However, the related studies are at the initial stage. In the future, to seek novel therapeutic targets, a large number of studies are needed to explore the specific role of lncRNAs in AP-associated organ injury.

## 5 LncRNAs as the potential biomarkers for the diagnosis of AP

In patients with AP, it is mild and moderate in approximately 80% of them, but 20% of them develop SAP. The mortality rate of 20% of patients with SAP can be as high as 30%, leading to serious medical and social burden worldwide (Garg and Singh, 2019; Lee and Papachristou, 2019; Trikudanathan et al., 2019). Therefore, to improve the prevention, management, and prognosis of patients with SAP, it is essential to identify novel potential biomarkers to predict the occurrence and mortality of SAP. Given the important role of inflammatory response in AP to SAP, some lncRNAs associated with inflammation are selected to explore their relativity with disease severity. LncRNA TSN1-2 is correlated with many inflammation-related diseases, such as sepsis, rheumatoid arthritis, and coronary artery disease (Gong et al., 2017; Xu and Shao, 2018; Zeng et al., 2019). Therefore, Li et al. (2020) explored its predictive value for SAP risk and investigated its correlation with disease severity and in-hospital mortality in SAP patients. In their study, the data were from 60 SAP, 60 moderate–severe acute pancreatitis (MSAP), 60 mild AP patients, and 60 healthy controls. They found that lncRNA TSN1-2 displayed a good predictive value for disease severity in SAP patients and for increased in-hospital mortality in SAP and MSAP patients, suggesting that lncRNA ITSN1-2 might be a potential biomarker to improve the prognosis of SAP patients. In another study, Liu and Lin (2022) found that lnc-PVT1 is associated with the CRP level, sequential organ failure assessment score, and higher mortality risk in AP patients, especially in SAP patients, suggesting its potential as a biomarker for AP. In a simple study, Wang et al. investigated the correlation of lncRNA B3GALT5-AS1 with the severity of this disease in 30 patients with SAP, 36 patients with MAP, and 28 healthy volunteers. They found the lncRNA B3GALT5-AS1 expression in patients with SAP was significantly lower than that in patients with MAP, suggesting that lncRNA B3GALT5-AS1 might be correlated with the severity of this disease (Wang et al., 2019). These studies indicated the potential of lncRNAs as novel biomarkers to predict and diagnose SAP for therapy and management. However, further validation with large populations from multiple regions is needed to investigate their sensitivity and specificity.

## 6 LncRNAs as the targets for AP therapy

Based on the above analysis, we confirmed that lncRNAs play a critical role in the occurrence and development of AP by being involved in its pathophysiological processes. Thus, manipulating the function of lncRNAs with inhibitors or mimics is a potential strategy for research on how to treat AP. In recent studies about traditional Chinese medicine and mesenchymal stem cells (MSCs) to treat AP, lncRNAs have been found to be the indirect targets for treating AP.

Emodin is a natural product that originates from *Rheum palmatum*. Previous studies have reported that emodin had good therapeutic effects on AP and significantly reduced mortality in SAP rats through inhibiting inflammatory response (Yao et al., 2015; Wang et al., 2016; Gao Z. et al., 2020). However, the specific therapeutic mechanism remains unclear. A recent study reported that lncRNA TUG1 was the therapeutic target for treating AP (Wen et al., 2021). In AR42J cells treated with caerulein and LPS, lncRNA TUG1 was upregulated, and emodin could inhibit the expression of lncRNA TUG1 and decrease the inflammatory level. The overexpression of lncRNA TUG1 could induce apoptosis and reverse the therapeutic effects of emodin. In addition, emodin could increase the anti-inflammatory function of regulatory T cells by inhibiting lncRNA TUG1. In another study, Xu et al. (2021) reported that emodin could relieve SAP-induced acute lung injury by regulating the expression of lncRNAs via affecting inflammatory and immune response pathways, based on high-throughput sequencing of lncRNAs and analysis in the rat model of SAP. However, the mechanisms in detail of these altered lncRNAs should be further explored.

In AP, another traditional Chinese medicine quercetin, which possesses anti-inflammation activity, has confirmed that quercetin can improve pathological changes, including inhibition of inflammatory responses and reduction of ER stress (Seo et al., 2019; Zhang et al., 2019). Sheng et al. (2021) found that quercetin could inhibit the p38/MAPK signaling pathway through upregulating miR-216b, thereby improving the inflammatory responses of AP. In this progress, miR-216b was the core molecule, which was the direct target of MAP2K6 of the p38/MAPK signaling pathway. Moreover, lncRNA NEAT1 was a direct target of miR-216b and could be suppressed by quercetin. Thus, lncRNA NEAT1 was identified as the direct target of quercetin to treat AP. In addition, in the AP model of mice and AR42J cells treated with baicalin, lncRNA MALAT1 was identified as a direct target of miR-15a to regulate MAP2K4, thereby inhibiting the JNK signaling pathway (Zhen et al., 2021).

MSC transplantation into SAP animals has shown good therapeutic effects due to its properties of low immunogenicity and immunomodulatory effect (Goodman et al., 2020; Huang et al., 2021). However, the related therapeutic mechanism remains unclear. Song et al. (2020) reported that lncRNA H19 was upregulated in rats receiving MSCs, and the overexpression of lncRNA H19 in MSCs significantly enhanced the anti-inflammatory capacity and inhibited autophagy in rats with SAP. Mechanically, lncRNA H19 enhanced the expression of protein tyrosine kinase 2 (PTK2, encoding FAK) to suppress autophagy by sponging miR-138-5p and enhanced the level of β-catenin to promote cell proliferation by sponging miR-141-3p.

## 7 Conclusion and perspective

To date, a large number of studies have revealed features and functions of lncRNAs and their key roles in human development and disease progressions with the development of high-throughput sequencing technologies. However, the exploration of the role of lncRNAs in AP is only 5 years up to now. Predictably, despite the study to explore lncRNAs in AP at the initial stage, current studies have demonstrated that lncRNAs play an important role in the pathogenesis, diagnosis, and therapy of AP. In the pathogenesis of AP, the abnormal expression of lncRNAs can influence trypsinogen activation, autophagy, inflammatory response, and acinar cell death, and these pathological events can influence each other further, aggravating pancreatic injury and other organ injury. For example, on one hand, some lncRNAs upregulate the expression of HMGB1, leading to the activation of the TLR4/NF-κB pathway. On the other hand, these lncRNAs can induce M1 polarization of macrophages. These events can promote the inflammatory response together. In therapy, lncRNAs have been found to be the indirect targets of traditional Chinese medicine and MSCs to treat AP. However, there is a lack of studies to explore the potential of lncRNAs as the direct targets in AP. In general, this evidence provides new insight into understanding the cellular and molecular mechanisms of AP and seeks novel biomarkers for the diagnosis and therapeutic targets based on current reports.

However, a large number of studies are needed to explore the roles of lncRNAs in AP pathogenesis, diagnosis, and therapy. For instance, in addition to the aforementioned four pathological events, the roles of lncRNAs in other pathological events remain unclear, such as pathological calcium signaling, mitochondrial dysfunction, and endoplasmic reticulum stress. Lots of inflammatory cells are involved in the inflammation of AP, such as macrophages, T lymphocytes, and peripheral blood mononuclear cells. However, current studies only focus on macrophages. In addition, organ injury is a common complication of AP, especially SAP, but only one paper reported the role of lncRNAs in AP-related intestinal injury. On the potential biomarkers for the diagnosis of AP, further studies have to investigate their sensitivity and specificity based on lots of populations from different regions. Finally, based on the role of lncRNAs in AP pathogenesis, studying inhibitors or mimics to manipulate lncRNA function is essential to treat AP. However, there are many challenges and limitations to the clinical use of lncRNAs. For example, the reports on direct or mediated lncRNA regulation by targeted siRNA delivery and related techniques are few. Nanoparticles, siRNA conjugates, and virus-based vectors may be the directions to explore the aforementioned problems in the future. Thus, lncRNAs are newcomers in the field of gene regulation, but therapeutic targeting of lncRNA will require a lot of additional studies. In the future, there is a long way to go to overcome and solve these challenges and questions before clinical usage of lncRNAs. By the way, exploring the inhibitor of downstream proteins of lncRNAs may be a good option. Based on the above analysis, we believe that our insights into lncRNAs will further deepen researchers’ and clinicians’ understanding of the pathogenesis of AP, providing a theoretical basis and new ideas for AP diagnosis and treatment and, finally, benefit patients.
